# Crystal structure of 4-meth­oxy-*N*-phenyl­benzamide

**DOI:** 10.1107/S1600536814016420

**Published:** 2014-08-01

**Authors:** Zhijun Wang, Haiying Lei, Linhua Jin, Ruitao Zhu

**Affiliations:** a Department of Chemistry, Changzhi University, Changzhi 046011, People’s Republic of China; b Department of Biological Science and Technology, Changzhi University, Changzhi 046011, People’s Republic of China; c Department of Chemistry, Taiyuan Normal University, Taiyuan 030031, People’s Republic of China

**Keywords:** crystal structure, amide, *C*(4) chain, hydrogen bonding

## Abstract

In the title mol­ecule, C_14_H_13_NO_2_, the dihedral angle between the planes of the benzene rings is 65.18 (4)°. The central amide group has about the same degree of twist with respect to both ring planes, as indicated by the dihedral angles of 34.70 (8) and 30.62 (8)° between its plane and that of the phenyl and 4-meth­oxy­benzene rings, respectively. The C atom of the meth­oxy group is close to being coplanar with its attached ring [deviation = −0.112 (2) Å]. In the crystal, mol­ecules are linked by inter-amide N—H⋯O hydrogen bonds, which generate *C*(4) chains propagating in the [100] direction. Adajcent mol­ecules in the chain are related by translational symmetry.

## Related literature   

The background to this work has been described in earlier papers; see: Ren *et al.* (2010 ▶); Zhu *et al.* (2011 ▶). For related structures, see: Raza *et al.* (2010 ▶); Gowda *et al.* (2003 ▶).
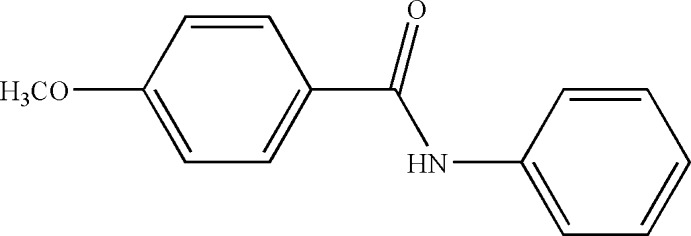


## Experimental   

### Crystal data  

C_14_H_13_NO_2_
*M* *_r_* = 227.25
Triclinic, 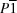

*a* = 5.308 (3) Å
*b* = 7.709 (4) Å
*c* = 14.109 (7) Å
α = 96.911 (8)°
β = 99.210 (8)°
γ = 90.511 (9)°
*V* = 565.5 (5) Å^3^
*Z* = 2
Mo *K*α radiation
μ = 0.09 mm^−1^
*T* = 296 K
0.30 × 0.20 × 0.20 mm

### Data collection  

Bruker SMART CCD diffractometer
Absorption correction: multi-scan (*SADABS*; Bruker, 2007 ▶) *T*_min_ = 0.974, *T*_max_ = 0.982
3188 measured reflections
2005 independent reflections
1605 reflections with *I* > 2σ(*I*)
*R*_int_ = 0.013

### Refinement  

*R*[*F* ^2^ > 2σ(*F* ^2^)] = 0.036
*wR*(*F* ^2^) = 0.097
*S* = 1.03
2005 reflections
156 parameters
H-atom parameters constrained
Δρ_max_ = 0.16 e Å^−3^
Δρ_min_ = −0.13 e Å^−3^

### 

Data collection: *SMART* (Bruker, 2007 ▶); cell refinement: *SAINT* (Bruker, 2007 ▶); data reduction: *SAINT*; program(s) used to solve structure: *SHELXS97* (Sheldrick, 2008 ▶); program(s) used to refine structure: *SHELXL97* (Sheldrick, 2008 ▶); molecular graphics: *SHELXTL* (Sheldrick, 2008 ▶); software used to prepare material for publication: *SHELXTL* and *PLATON* (Spek, 2009 ▶).

## Supplementary Material

Crystal structure: contains datablock(s) I, New_Global_Publ_Block. DOI: 10.1107/S1600536814016420/hb7253sup1.cif

Structure factors: contains datablock(s) I. DOI: 10.1107/S1600536814016420/hb7253Isup2.hkl

Supporting information file. DOI: 10.1107/S1600536814016420/hb7253Isup3.cml

**Fig. 1.**
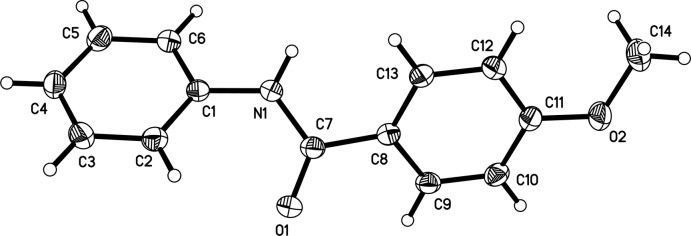
The molecular structure of the title compound with displacement ellipsoids drawn at the 30% probability level.

. DOI: 10.1107/S1600536814016420/hb7253fig1.tif
The mol­ecular structure of the title compound with displacement ellipsoids drawn at the 30% probability level.

**Fig. 2.**
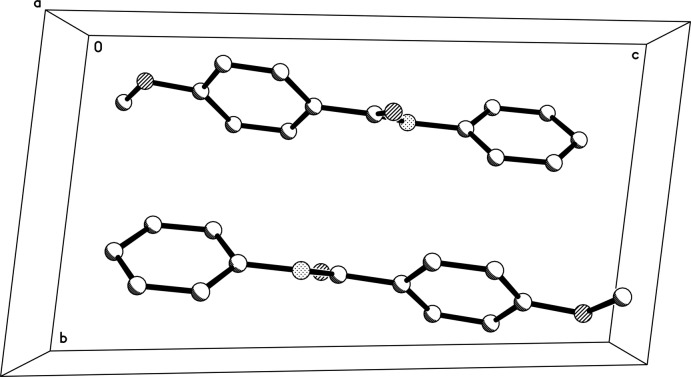
Part of the crystal structure of (I) with the donor-acceptor distances of hydrogen bonds drawn as dashed lines. H atoms are not shown.

. DOI: 10.1107/S1600536814016420/hb7253fig2.tif
Part of the crystal structure of (I) with the donor-acceptor distances of hydrogen bonds drawn as dashed lines. H atoms are not shown.

CCDC reference: 1014108

Additional supporting information:  crystallographic information; 3D view; checkCIF report

## Figures and Tables

**Table 1:** Hydrogen-bond geometry (Å, °)

*D*—H⋯*A*	*D*—H	H⋯*A*	*D*⋯*A*	*D*—H⋯*A*
N1—H1⋯O1^i^	0.86	2.31	3.110 (2)	154

Symmetry code: (i) 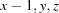.

## supplementary crystallographic information

### S1. Experimental

To a 100 ml round flask fitted with a condenser was added aniline (0.93 g, 10 mmol), dichloromethane (15 ml) and triethylamine(0.5 ml) with magnetic stirring. 4-methoxybenzoyl chloride (1.70 g, 10 mmol) was added gradually. The reaction mixture was stirred at room temperature for 1 h and then refluxed for 2 h. The product precipitated as a white powder, which was washed three times with water and dichloromethane. Recrystallization from ethyl alcohol solution produced colourless prisms of the title compound.

### S2. Refinement

H atoms were placed in idealized positions and allowed to ride on their respective parent atoms, with C—H = 0.93 Å, N—H = 0.86 Å and *U*_iso_(H)= 1.2*U*_eq_(C,*N*).

### Crystal data

**Table:**

C_14_H_13_NO_2_	*V* = 565.5 (5) Å^3^
*M**_r_* = 227.25	*Z* = 2
Triclinic, *P*1	*F*(000) = 240
Hall symbol: -P 1	*D*_x_ = 1.335 Mg m^−^^3^
*a* = 5.308 (3) Å	Mo *K*α radiation, λ = 0.71073 Å
*b* = 7.709 (4) Å	θ = 1.5–25.1°
*c* = 14.109 (7) Å	µ = 0.09 mm^−^^1^
α = 96.911 (8)°	*T* = 296 K
β = 99.210 (8)°	Prism, colorless
γ = 90.511 (9)°	0.30 × 0.20 × 0.20 mm

### Data collection

**Table:**

Bruker SMART CCD diffractometer	2005 independent reflections
Radiation source: fine-focus sealed tube	1605 reflections with *I* > 2σ(*I*)
Graphite monochromator	*R*_int_ = 0.013
ω scans	θ_max_ = 25.1°, θ_min_ = 1.5°
Absorption correction: multi-scan (*SADABS*; Bruker, 2007)	*h* = −5→6
*T*_min_ = 0.974, *T*_max_ = 0.982	*k* = −8→9
3188 measured reflections	*l* = −16→16

### Refinement

**Table:**

Refinement on *F*^2^	Secondary atom site location: difference Fourier map
Least-squares matrix: full	Hydrogen site location: inferred from neighbouring sites
*R*[*F*^2^ > 2σ(*F*^2^)] = 0.036	H-atom parameters constrained
*wR*(*F*^2^) = 0.097	*w* = 1/[σ^2^(*F*_o_^2^) + (0.045*P*)^2^ + 0.1154*P*] where *P* = (*F*_o_^2^ + 2*F*_c_^2^)/3
*S* = 1.03	(Δ/σ)_max_ < 0.001
2005 reflections	Δρ_max_ = 0.16 e Å^−^^3^
156 parameters	Δρ_min_ = −0.13 e Å^−^^3^
0 restraints	Extinction correction: *SHELXL97* (Sheldrick, 2008), Fc^*^=kFc[1+0.001xFc^2^λ^3^/sin(2θ)]^-1/4^
Primary atom site location: structure-invariant direct methods	Extinction coefficient: 0.037 (5)

### Special details

**Table:**

Geometry. All e.s.d.'s (except the e.s.d. in the dihedral angle between two l.s. planes) are estimated using the full covariance matrix. The cell e.s.d.'s are taken into account individually in the estimation of e.s.d.'s in distances, angles and torsion angles; correlations between e.s.d.'s in cell parameters are only used when they are defined by crystal symmetry. An approximate (isotropic) treatment of cell e.s.d.'s is used for estimating e.s.d.'s involving l.s. planes.
Refinement. Refinement of *F*^2^ against ALL reflections. The weighted *R*-factor *wR* and goodness of fit *S* are based on *F*^2^, conventional *R*-factors *R* are based on *F*, with *F* set to zero for negative *F*^2^. The threshold expression of *F*^2^ > σ(*F*^2^) is used only for calculating *R*-factors(gt) *etc*. and is not relevant to the choice of reflections for refinement. *R*-factors based on *F*^2^ are statistically about twice as large as those based on *F*, and *R*- factors based on ALL data will be even larger.

### Fractional atomic coordinates and isotropic or equivalent isotropic displacement parameters (Å^2^)

**Table:**

	*x*	*y*	*z*	*U*_iso_*/*U*_eq_	
N1	0.2321 (2)	0.27538 (17)	0.57341 (8)	0.0387 (3)	
H1	0.0748	0.2753	0.5465	0.046*	
O1	0.63875 (19)	0.25639 (17)	0.54382 (8)	0.0542 (4)	
O2	0.0921 (2)	0.14685 (16)	0.11034 (7)	0.0520 (3)	
C1	0.2818 (3)	0.29568 (19)	0.67586 (10)	0.0340 (3)	
C2	0.4919 (3)	0.3893 (2)	0.72863 (11)	0.0427 (4)	
H2	0.6085	0.4409	0.6971	0.051*	
C3	0.5279 (3)	0.4060 (2)	0.82849 (12)	0.0504 (4)	
H3	0.6693	0.4693	0.8638	0.060*	
C4	0.3582 (3)	0.3307 (2)	0.87637 (11)	0.0483 (4)	
H4	0.3849	0.3414	0.9436	0.058*	
C5	0.1478 (3)	0.2391 (2)	0.82331 (11)	0.0468 (4)	
H5	0.0306	0.1889	0.8551	0.056*	
C6	0.1091 (3)	0.2210 (2)	0.72366 (11)	0.0401 (4)	
H6	−0.0333	0.1585	0.6886	0.048*	
C7	0.4093 (3)	0.2561 (2)	0.51365 (11)	0.0373 (4)	
C8	0.3083 (3)	0.22995 (19)	0.40820 (10)	0.0345 (3)	
C9	0.4522 (3)	0.1323 (2)	0.34765 (11)	0.0390 (4)	
H9	0.6042	0.0850	0.3742	0.047*	
C10	0.3727 (3)	0.1051 (2)	0.24959 (11)	0.0415 (4)	
H10	0.4676	0.0365	0.2103	0.050*	
C11	0.1516 (3)	0.1795 (2)	0.20900 (10)	0.0377 (4)	
C12	0.0070 (3)	0.2788 (2)	0.26739 (11)	0.0395 (4)	
H12	−0.1412	0.3297	0.2403	0.047*	
C13	0.0852 (3)	0.3014 (2)	0.36659 (10)	0.0378 (4)	
H13	−0.0139	0.3658	0.4060	0.045*	
C14	−0.1445 (4)	0.2052 (3)	0.06486 (12)	0.0631 (5)	
H14A	−0.2804	0.1623	0.0940	0.095*	
H14B	−0.1707	0.1620	−0.0028	0.095*	
H14C	−0.1425	0.3307	0.0726	0.095*	

### Atomic displacement parameters (Å^2^)

**Table:**

	*U*^11^	*U*^22^	*U*^33^	*U*^12^	*U*^13^	*U*^23^
N1	0.0288 (6)	0.0535 (8)	0.0333 (7)	0.0023 (5)	0.0034 (5)	0.0053 (6)
O1	0.0299 (6)	0.0892 (10)	0.0424 (7)	0.0060 (6)	0.0046 (5)	0.0050 (6)
O2	0.0563 (7)	0.0653 (8)	0.0327 (6)	0.0104 (6)	0.0067 (5)	0.0000 (5)
C1	0.0313 (7)	0.0364 (8)	0.0342 (8)	0.0065 (6)	0.0052 (6)	0.0035 (6)
C2	0.0360 (8)	0.0479 (10)	0.0434 (9)	−0.0027 (7)	0.0083 (7)	−0.0002 (7)
C3	0.0418 (9)	0.0583 (11)	0.0453 (10)	0.0005 (8)	0.0001 (7)	−0.0073 (8)
C4	0.0520 (10)	0.0587 (11)	0.0325 (8)	0.0115 (8)	0.0045 (7)	0.0016 (7)
C5	0.0471 (9)	0.0546 (11)	0.0418 (9)	0.0046 (8)	0.0140 (7)	0.0088 (8)
C6	0.0333 (8)	0.0477 (9)	0.0387 (8)	−0.0004 (7)	0.0055 (6)	0.0036 (7)
C7	0.0309 (8)	0.0416 (9)	0.0399 (8)	0.0030 (6)	0.0059 (6)	0.0066 (7)
C8	0.0314 (7)	0.0370 (8)	0.0358 (8)	−0.0006 (6)	0.0072 (6)	0.0051 (6)
C9	0.0313 (8)	0.0427 (9)	0.0437 (9)	0.0050 (6)	0.0072 (6)	0.0059 (7)
C10	0.0400 (8)	0.0446 (9)	0.0411 (9)	0.0060 (7)	0.0142 (7)	−0.0004 (7)
C11	0.0403 (8)	0.0390 (9)	0.0343 (8)	−0.0009 (7)	0.0087 (6)	0.0033 (6)
C12	0.0348 (8)	0.0451 (9)	0.0379 (8)	0.0062 (7)	0.0035 (6)	0.0052 (7)
C13	0.0357 (8)	0.0408 (9)	0.0372 (8)	0.0062 (6)	0.0094 (6)	0.0010 (7)
C14	0.0676 (12)	0.0819 (14)	0.0369 (9)	0.0150 (10)	0.0004 (8)	0.0059 (9)

### Geometric parameters (Å, º)

**Table:**

N1—C7	1.3580 (19)	C6—H6	0.9300
N1—C1	1.4164 (19)	C7—C8	1.487 (2)
N1—H1	0.8600	C8—C13	1.385 (2)
O1—C7	1.2242 (18)	C8—C9	1.394 (2)
O2—C11	1.3689 (18)	C9—C10	1.370 (2)
O2—C14	1.419 (2)	C9—H9	0.9300
C1—C2	1.382 (2)	C10—C11	1.382 (2)
C1—C6	1.381 (2)	C10—H10	0.9300
C2—C3	1.381 (2)	C11—C12	1.384 (2)
C2—H2	0.9300	C12—C13	1.384 (2)
C3—C4	1.373 (2)	C12—H12	0.9300
C3—H3	0.9300	C13—H13	0.9300
C4—C5	1.378 (2)	C14—H14A	0.9600
C4—H4	0.9300	C14—H14B	0.9600
C5—C6	1.378 (2)	C14—H14C	0.9600
C5—H5	0.9300		
			
C7—N1—C1	126.16 (12)	C13—C8—C9	118.33 (14)
C7—N1—H1	116.9	C13—C8—C7	123.93 (13)
C1—N1—H1	116.9	C9—C8—C7	117.71 (13)
C11—O2—C14	118.22 (13)	C10—C9—C8	120.86 (14)
C2—C1—C6	119.58 (14)	C10—C9—H9	119.6
C2—C1—N1	122.48 (13)	C8—C9—H9	119.6
C6—C1—N1	117.92 (13)	C9—C10—C11	120.12 (14)
C1—C2—C3	119.65 (15)	C9—C10—H10	119.9
C1—C2—H2	120.2	C11—C10—H10	119.9
C3—C2—H2	120.2	O2—C11—C10	115.38 (13)
C4—C3—C2	121.03 (16)	O2—C11—C12	124.49 (14)
C4—C3—H3	119.5	C10—C11—C12	120.13 (14)
C2—C3—H3	119.5	C13—C12—C11	119.30 (14)
C3—C4—C5	119.01 (15)	C13—C12—H12	120.4
C3—C4—H4	120.5	C11—C12—H12	120.4
C5—C4—H4	120.5	C12—C13—C8	121.22 (14)
C4—C5—C6	120.70 (15)	C12—C13—H13	119.4
C4—C5—H5	119.6	C8—C13—H13	119.4
C6—C5—H5	119.6	O2—C14—H14A	109.5
C5—C6—C1	120.02 (15)	O2—C14—H14B	109.5
C5—C6—H6	120.0	H14A—C14—H14B	109.5
C1—C6—H6	120.0	O2—C14—H14C	109.5
O1—C7—N1	122.62 (14)	H14A—C14—H14C	109.5
O1—C7—C8	121.34 (13)	H14B—C14—H14C	109.5
N1—C7—C8	116.02 (13)		

### Hydrogen-bond geometry (Å, º)

**Table:**

*D*—H···*A*	*D*—H	H···*A*	*D*···*A*	*D*—H···*A*
N1—H1···O1^i^	0.86	2.31	3.110 (2)	154

Symmetry code: (i) *x*−1, *y*, *z*.
