# Supplementary figures and images for: Crystal structure of 4-meth­oxy-N-phenyl­benzamide
Source: Acta Crystallogr Sect E Struct Rep Online. 2014 Aug 1;70(Pt 9):o921. doi: 10.1107/S1600536814016420 (PMC4186206; doi:10.1107/S1600536814016420)

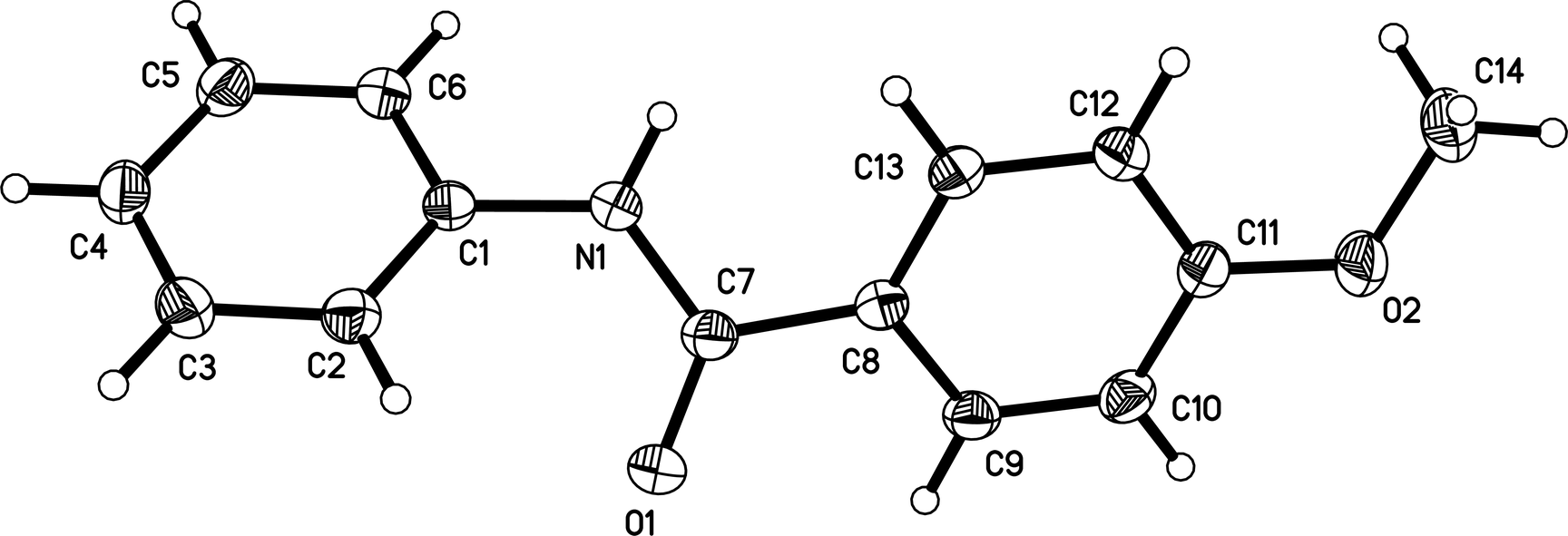

Supplement: Supplementary file 4 [file e-70-0o921-fig1.tif]

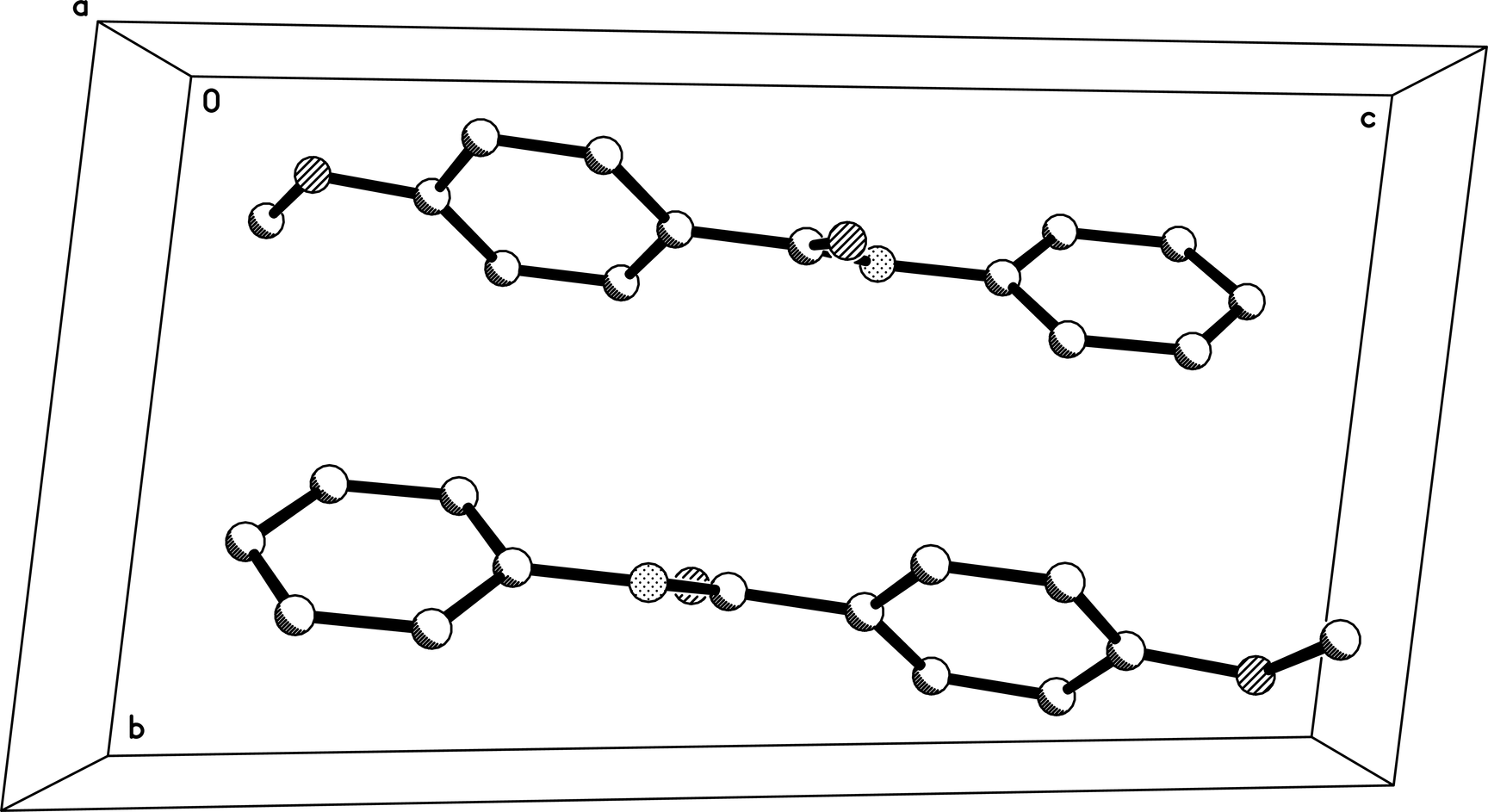

Supplement: Supplementary file 5 [file e-70-0o921-fig2.tif]
